# Lymphocyte-To-Monocyte Ratio as the Best Simple Predictor of Bacterial Infection in Patients with Liver Cirrhosis

**DOI:** 10.3390/ijerph17051727

**Published:** 2020-03-06

**Authors:** Damian Piotrowski, Anna Sączewska-Piotrowska, Jerzy Jaroszewicz, Anna Boroń-Kaczmarska

**Affiliations:** 1 Department of Infectious Diseases and Hepatology, Medical University of Silesia, 40-055 Katowice, Poland; jjaroszewicz@sum.edu.pl; 2 Department of Labour Market Research and Forecasting, University of Economics, 40-287 Katowice, Poland; anna.saczewska-piotrowska@ue.katowice.pl; 3 Department of Infectious Diseases, Andrzej Frycz Modrzewski Krakow University, 30-705 Krakow, Poland; annabk@inet.com.pl

**Keywords:** liver cirrhosis, bacterial infections, community-acquired infections, nosocomial infections, risk assessment, LMR, logistic regression

## Abstract

*Background and aim*: The aim of this study was to assess the diagnostic performance of new morphology-related indices and Child–Turcotte–Pugh (CTP) and Model for End-Stage Liver Disease (MELD) scores during hospitalization in predicting the onset of bacterial infection in patients with liver cirrhosis. *Material and methods*: A total of 171 patients (56.9% males; median age 59 years; total number of hospitalizations 209) with liver cirrhosis were included in this observational study. The diagnosis of cirrhosis was made on the basis of clinical, biochemical, ultrasonic, histological, and endoscopic findings. The neutrophil-to-lymphocyte ratio (NLR), lymphocyte-to-monocyte ratio (LMR), modified aspartate aminotransferase-to-platelet ratio index (APRI), aspartate aminotransferase-to-alanine aminotransferase ratio (AAR), Fibrosis-4 index (FIB-4), platelet-to-lymphocyte ratio (PLR), neutrophil-to-monocyte ratio (NMR), and CTP and MELD scores were calculated for the cases of patients with cirrhosis. *Results:* Bacterial infection was diagnosed in 60 of the 209 (28.7%) hospitalizations of patients with cirrhosis. The most common infections were urinary tract infection (UTI), followed by pneumonia and sepsis. The more severe the liver failure, the greater the bacterial infection prevalence and mortality. Patients with decompensated liver cirrhosis were infected more often than subjects with compensated cirrhosis (50.0% vs. 12.9%, *p* = 0.003). The calculated MELD score, CTP, NLR, LMR, AAR, monocyte count, and C-reactive protein (CRP) concentration were also related to the bacterial infection prevalence, and mortality areas under the curve (AUC) were 0.629, 0.687, 0.606, 0.715, 0.610, 0.648, and 0.685, respectively. The combined model with two variables (LMR and CTP) had the best AUC of 0.757. The most common bacteria isolated from patients with UTI were *Escherichia coli*, *Enterococcus faecalis*, and *Klebsiella pneumonia*. Gram-negative bacteria were also responsible for spontaneous bacterial peritonitis (SBP), and together with gram-positive *streptococci* and *staphylococci*, these microorganisms were isolated from blood cultures of patients with sepsis. Significant differences were found between CTP classification, MELD score, NLR, LMR, AAR, CRP, and PLR in patients with cirrhosis with, or without, bacterial infection. *Conclusions:* Bacterial infection prevalence is relatively high in patients with liver cirrhosis. Although all analyzed scores, including the LMR, NLR, aspartate aminotransferase (AST)/alanine aminotransferase (ALT), CRP, CTP, and MELD, allowed the prediction of bacterial occurrence, the LMR had the highest clinical utility, according to the area under the curve (AUC) and odds ratio (OR).

## 1. Introduction

Liver damage may be caused by various factors, including, but not limited to, viral infections, toxic damage, and autoimmune and metabolic diseases. Liver cirrhosis is the final stage of the damage sustained by this organ [1]. Studies regarding liver cirrhosis show that this group of patients is more vulnerable to bacterial infection [2,3], and its probability after admission is 25–35% [4], which is 4–5-fold higher than the risk for the general population. Urinary tract infection, pneumonia, spontaneous bacterial peritonitis (SBP), and sepsis are the most common types of infection in cirrhosis. However, the proportions of these infections vary in different studies worldwide.

Due to the higher infection occurrence in patients with decompensated liver cirrhosis, the scores that predict the outcome in patients with cirrhosis were taken into consideration, as to whether they can also predict bacterial infection occurrence. Additionally, inflammatory markers were considered to be associated with the prognosis of bacterial infection in liver cirrhosis. This study describes the potential use of the neutrophil-to-lymphocyte ratio (NLR), lymphocyte-to-monocyte ratio (LMR), modified aspartate aminotransferase-to-platelet ratio index (APRI), Fibrosis-4 index (FIB-4), platelet-to-lymphocyte ratio (PLR), and neutrophil-to-monocyte ratio (NMR), which are cost-effective, easily accessible markers of infection. The NLR was considered a good predictor of hospital-acquired (HA) bacterial infections in decompensated liver cirrhosis by Cai et al. [5]. The NLR, LMR, and Model for End-Stage Liver Disease (MELD) score were considered as predictors for early recurrence of hepatocellular carcinoma after transarterial chemoembolization (Elalfy et al. [6]). Zeng et al. [7] studied the aspartate aminotransferase-to-alanine aminotransferase ratio (AAR), APRI, FIB-4, NLR, LMR, and PLR for monitoring the course of autoimmune hepatitis. All morphology-related markers were described for the first time as markers of inflammation or as predictors in neoplastic diseases [8,9,10,11].

To assess the severity of liver failure, two score models are commonly used. The first score is the Child–Turcotte–Pugh (CTP) score, initially proposed by Child and Turcotte and modified by Pugh et al. [12]. The second is the Model for End-Stage Liver Disease [13]. These two models are widely used to predict the outcomes of patients with cirrhosis.

The aim of this manuscript is to present the results of a retrospective analysis of hospitalizations of patients with cirrhosis in a single university center in order to confirm the predictive value of simple morphology and biochemistry-derived markers and the CTP and MELD scores for the incidence of bacterial infections.

## 2. Materials and Methods 

Study design: A total of 209 discharge cards from hospitalizations between 2004 and 2019 in a single university center were selected for this observational study. The only inclusion criterion was the diagnosis of liver cirrhosis based on clinical, biochemical, ultrasound, histological, and endoscopic findings. The total number of patients included was 171 (56.9% males; median age 59 years). Most of them were hospitalized once in the studied period of time; 15 patients were admitted twice; four patients three times; two patients were hospitalized four times; and another two patients five times. The study was approved by the Ethics Committee of the Medical University of Silesia.

Data collection: The total number of discharge cards was 5074. Among them, 209 fulfilled the inclusion criterion for the study group. The discharge cards were analyzed for infections. Routine laboratory parameters and clinical findings were used to calculate the NLR, LMR, AAR, APRI, FIB-4, PLR, and NMR, as well as the MELD score and Child–Pugh classification.

Prognostic scores: The samples for testing were collected from patients within 24 h after admission. Formulae to calculate predictors are presented in Table 1.

Definition and diagnosis: The definition of primary SBP covers an infection of the ascitic fluid that was formerly sterile with no intra-abdominal infection source [3]. The diagnosis of SBP was confirmed when the polymorphonuclear (PMN) count in the ascitic fluid was greater than or equal to 250 cells/mm^3^ [4,17,18].

According to Bonkat et al. [19], the diagnosis of UTI can be made when more than 10 leucocytes can be found in 1 mm^3^ of a urine sample or more than 10^5^ colony forming units (CFU) exist in a single milliliter of urine in two consecutive cultures that take place more than 24 h apart.

Diagnosis of pneumonia is based on a constellation of suggestive clinical features, along with infiltration in the lungs shown by chest X-ray or other imaging techniques, with or without supporting microbiological data [20].

In cases where the patient’s calculated sepsis-related organ failure assessment (SOFA) score increased suddenly by two points, and blood cultures were positive, sepsis was diagnosed.

Community-acquired (CA) infection was defined as an infection set in a patient who did not use healthcare facilities or where infection was present within the first two days of hospitalization [21]. The healthcare-associated infection definition covers patients infected within the first 48 h of hospitalization, combined with healthcare contact (hospitalization for at least two days, intravenous chemotherapy within one month before infection, surgery in the past six months, or residence in a nursing home or a long-term care facility) [22]. Nosocomial infection was defined as an infection diagnosed two days after admission to the hospital [23].

Statistical methods: All statistical analyses were calculated using STATISTICA [24] and R [25] with gmodels [26], OptimalCutpoints [27], and verification (NCAR–Research Applications Laboratory) [28] packages.

Continuous variables were analyzed using the Shapiro–Wilk tests to determine whether they followed a normal distribution. Student’s t-test and the Mann–Whitney U test were used depending on the variables’ situation, that is, whether or not they were normally distributed. Receiver operating characteristic (ROC) curve analysis was performed. Univariate and multivariate logistic (forward and backward stepwise) regression analyses were performed to explore risk factors associated with the presence of bacterial infection in patients with liver cirrhosis. In the models, odds ratios (OR) and 95% confidence intervals (CI) were computed. The area under the ROC curve (AUC) was used to measure the accuracy. The optimum cutoff point was identified using the Youden index. For the optimal cutoff point, the sensitivity (Se), specificity (Sp), positive predictive value (PPV), and negative predictive value (NPV) were calculated. The factors were considered significant when the *p*-value was less than 0.05.

## 3. Results

The patients who were included in this study had a diagnosis of liver cirrhosis. The diagnosis was based on clinical, biochemical, ultrasound, histological, and endoscopic findings and results. The reasons for the onset of liver cirrhosis varied; however, most of the analyzed patients were cirrhotic due to viral infections: HCV infection was the basis of liver cirrhosis in 59.3%, HBV infection in 13.9%, and alcohol liver cirrhosis in 11.0%. The remaining cirrhosis cases were caused by autoimmune hepatitis, primary biliary cirrhosis, nonalcoholic steatohepatitis, or the origin of the disease remained unknown.

The number of deceased patients within this group was 29 (13.88%). The infection within this group was diagnosed in 13 cases (44.8%), and occurrence of infection in patients who survived and were discharged (180 patients) was 26.1% (the difference in infection rate between these two groups was significant; *p* = 0.039). Among admitted patients, different infections were diagnosed. The total number of infections diagnosed was 60, and the most common was UTI (20.1%), followed by pneumonia (3.8%), sepsis (3.4%), SBP (2.4%), abscess of subcutaneous tissue (1.0%), infected post-operative wound (0.5%), and borreliosis (0.5%). It is also worth noting that 54 patients had one infection source, but three had both UTI and sepsis, one had both pneumonia and sepsis, one had both skin abscess and sepsis, and in one case, UTI, SBP, and sepsis were diagnosed. Nosocomial infection was diagnosed in seven cases, healthcare-associated infection in 28 cases, and the remaining 25 cases of infections did not fulfill nosocomial or healthcare-associated infection; these were considered as community-acquired. A total of 58 infected patients were hospitalized once, and two cases of infection, which were the basis of liver cirrhosis decompensation, occurred in a single patient (the first infection in this patient was pneumonia; the second was UTI). 

The remaining 149 hospitalizations were urgent in 102 cases and involved patients with liver cirrhosis decompensation (the most common leading symptom was a presence of ascites, followed by encephalopathy and jaundice), kidney failure, and the need for quick diagnosis of hepatocellular carcinoma. The remaining 47 hospitalizations were scheduled (planned liver biopsy; qualification for antiviral treatment in hepatitis C virus (HCV) and hepatitis B virus (HBV) infected patients).

The attempt to find a relationship between specific bacteria, or specific infection type, and outcome showed a lack of statistical significance.

The pathogens that were found in the cultures (taking into account the type of infection) are specified in Table 2. 

It must be noted that the sputum cultures were negative in five cases (pneumonia was confirmed based on clinical features, along with infiltration in the lungs shown by chest X-ray). The probable reason was the fact that the patients were already treated with antibiotics before sputum collection. The total number of positive cultures was 67, which was greater than the number of infected patients, because some of the patients were diagnosed with UTI and sepsis (three cases), pneumonia and sepsis (one case), skin abscess and sepsis (one case), and one of the patients had UTI, SBP and sepsis.

The infections were divided into two groups: nosocomial and both healthcare-associated and community-acquired. There were only seven cases in which nosocomial infection was proven. The remaining 53 cases fell into the community-acquired or healthcare-associated group. The huge disproportion of these group sizes was the reason for the lack of statistical significance between them. 

Differences in clinical and laboratory assessments between patients with or without infection are presented in Table 3.

**Figure 1:**
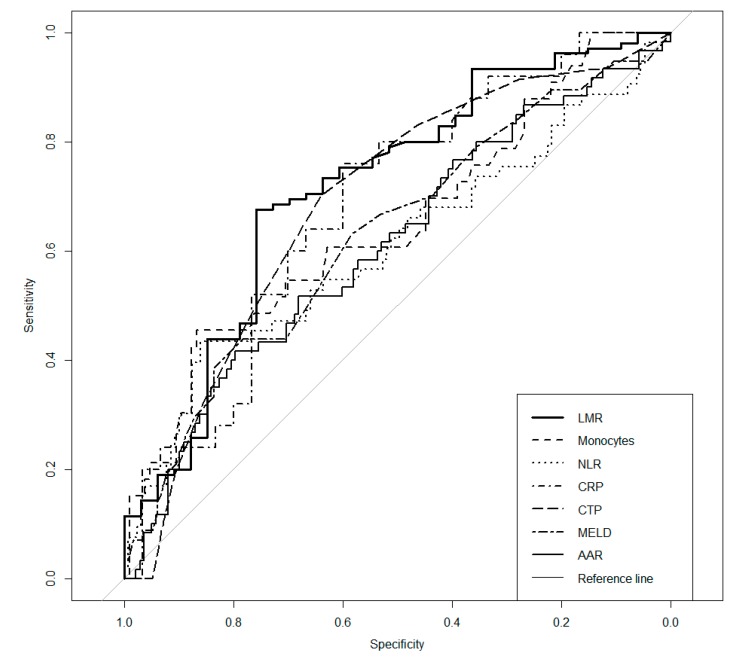
Receiver operating characteristics (ROC) curves. NLR—neutrophil-to-lymphocyte ratio; LMR—lymphocyte-to-monocyte ratio; AAR—aspartate aminotransferase-to-alanine aminotransferase ratio; CRP—C-reactive protein; CTP—Child-Turcotte-Pugh; MELD—Model for End-Stage Liver Disease.

The NLR, LMR, PLR, monocyte count, AAR, C-reactive protein (CRP), CTP, and MELD values were significantly different in the group of patients with bacterial infection, compared to those of noninfected patients. The LMR was the only destimulant, which means that lower values were more likely found in patients with infection; the remaining variables were higher in infected patients. The next step in the analysis was to define which of the tests is the best predictor of infection among patients with cirrhosis. To achieve this goal, ROC curves were calculated, and AUCs were compared. The highest AUC (0.715) was found for the LMR, which suggests that it is a good predictor of bacterial infection in patients with cirrhosis. Additionally, the CTP and MELD scoring, CRP concentration, monocyte count, AAR, and NLR had moderate abilities to discriminate patients with or without infection. ROC curves and AUCs with cut-offs are presented in Figure 1 and Table 4. Additionally, Se, Sp, PPV, and NPV were calculated for parameters that were statistically significant. The highest PPV was observed for the LMR (89.9%), which makes this predictor the best for screening patients for infection, while monocyte counts followed by the NLR, AAR, and CRP concentrations had the best NPV (83.5%, 78.7%, 75.9%, and 75%, respectively); therefore, they are able to exclude infection in patients (see Table 4).

Binary logistic regression analysis was performed to explore risk factors associated with the presence of bacterial infection in patients with liver cirrhosis. In univariate logistic regression analysis (see Table 5), the LMR, monocyte count, NLR, CRP, CTP, MELD, and AAR were associated with the presence of infection. 

In multivariate stepwise logistic regression (Table 5), the combined LMR and CTP were identified as also being associated with infection in patients with cirrhosis. The multivariate regression model with variables LMR and CTP had an AUC of 0.757 (*p* < 0.001). The ORs for variables included in this model were 0.22 (0.08–0.64; 95% CI) for LMR and 2.77 (1.01–7.60; 95% CI) for CTP. The cutoff point was calculated as 0.34 for this model, which signifies the classification threshold probability that is used to classify patients as non-infected (the probability less than this critical value) or with infection (the probability equal to or greater than the cutoff point) in a logistic regression. Based on this model, the following were calculated: Se (0.750), Sp (0.676), PPV (0.477), and NPV (0.873).

## 4. Discussion

Patients with cirrhosis have an increased risk of developing bacterial infections due to bacterial overgrowth and increased bacterial translocation [2,3]. Bacterial translocation (BT) is a physiological process in healthy conditions and is crucial for host immunity. Epithelial integrity is essential for preventing the entry of potentially harmful substances (bacteria or their products) into the portal circulation through transcellular or paracellular pathways [29]. A study by Pijls et al. showed increased intestinal permeability in patients with compensated cirrhosis, compared with that of the control group. Bacterial translocation also causes a proinflammatory state that worsens the hemodynamic status of patients with cirrhosis and leads to decompensation. Immune activation (“cytokine storm”) causes the development of cirrhosis-associated immune dysfunction that increases susceptibility to other infections [30,31,32].

Bacterial infection is an important problem in patients with liver cirrhosis. The presence of infection at admission, or during hospitalization, varies in different studies between 20% and 60% of patients [33]. The prevalence of infections from this study was 28.7%, which is in agreement with the published literature. According to the literature, the most common infections in patients with cirrhosis are SBP, UTI, pneumonia, bacteremia, and cellulitis. The most common types of bacterial infection in this study were UTI, pneumonia, SBP, and others.

The NLR has been described widely as a predictor of HA bacterial infections in decompensated cirrhosis [5], and it correlates with tumor necrosis factor alpha (TNF-α) and interleukin 6 (IL-6) concentrations [10]. It was introduced as a potential marker of inflammation, such as that associated with myocardial infarction [34,35], atherosclerosis, cystic fibrosis, diabetes mellitus, and infections [10,36,37,38,39,40,41]. The NLR and PLR were positively correlated with CRP concentration, while the LMR was negatively correlated with CRP concentration. Different authors have shown in their studies that in specific diseases, different morphology-derived markers were the most accurate. Turkmen et al. [10] showed that the PLR was better than the NLR in end-stage renal diseases. The LMR was not assessed in that study, and our results did not prove the advantage of the PLR over the LMR or NLR. In other studies, the LMR was found to be the best predictor for survival in patients with hepatocellular carcinoma (HCC) undergoing liver resection [42]. Among these novel inflammatory biomarkers, the LMR was characterized by the highest AUC and OR, which makes the LMR the best marker of infection in liver cirrhosis.

The LMR was also studied in patients with liver cirrhosis by Qi [43], Jamil and Durrani [44], and Cai et al. [45], but in these studies, the LMR was considered to be a marker of patient outcome, not as a marker for infection incidence. It must be mentioned that there are only a few studies regarding the LMR and liver cirrhosis, and among them, there is no evaluation of the LMR as a predictor of infection in patients with cirrhosis. This study is an attempt to fill this gap.

Both the CTP and MELD scores have the ability to discriminate between patients with a higher or lower risk of bacterial infection. The MELD score has a predictive value for both in-hospital mortality and bacterial infection incidence in patients with cirrhosis [46]. The CTP score is also known to have predictive significance in short-term and medium-term mortality evaluations in patients with liver cirrhosis [47]. The present study shows that both the CTP and MELD scores may also discriminate patients with a lower and higher risk of infection; however, the odds ratio and AUC for CTP and MELD scores are lower than those for the LMR.

The present study shows that the LMR, CTP, CRP, MELD, and NLR may be used to identify patients at greater risk of being infected. The optimal cut-offs for all markers have been evaluated. The current study shows that bacterial infection incidence in patients with liver cirrhosis should be considered, especially when the LMR is lower than 2.014. The other predictors (CTP higher than 9, CRP concentration greater than 9.2 mg/L, MELD score exceeding 18 points, or NLR higher than 3.957) had a lower AUC; however, they were still significantly associated with a higher bacterial infection risk. Therefore, these markers could be useful when considering an antimicrobial empiric treatment for patients with cirrhosis.

## 5. Conclusions

Bacterial infection prevalence is relatively high in patients with liver cirrhosis. Although all analyzed scores, including the LMR, NLR, AST/ALT, CRP, PLR, CTP, and MELD, allowed prediction of the bacterial occurrence, the LMR turned out to have the highest clinical utility according to the area under the curve and odds ratio.

## Figures and Tables

**Table 1:** Indices calculated using widely available parameters.

Calculated Indices	First Publication	Required Parameters	Formula
AAR	Williams, Hoofnagle (1988) [14]	AST, ALT	$\frac{AST}{ALT}$
Modified APRI	Fix et al. (2005) [15]	albumin, AST, PLT, age	$\frac{age\times AST\;}{albumin\;\left[\frac{g}{dL}\right]\times PLT\;\left[\frac{10^{9}}{L}\right]}$
FIB-4	Sterling et al. (2006) [16]	ALT, AST, PLT, age	$\frac{age\times AST}{PLT\;\left[\frac{10^{9}}{L}\right]\times \sqrt{ALT}}$
NLR	Zahorec R (2001) [8]	neutrophil, lymphocyte	$\frac{neutrophil}{lymphocyte}$
LMR	Merekoulias G et al. (2010) [9]	lymphocyte, monocyte	$\frac{lymphocyte}{monocyte}$
PLR	Turkmen K et al. (2013) [10]	PLT, lymphocyte	$\frac{PLT\;\left[\frac{10^{9}}{L}\right]}{lymphocyte}$
NMR	Cihan YB et al. (2013) [11]	neutrophil, monocyte	$\frac{neutrophil}{monocyte}$
CTP	Pugh et al. (1973) [12]	bilirubin, albumin, prothrombin time, ascites, encephalopathy	^a^
MELD	Bambha K et al. (2004) [13]	creatinine, bilirubin, INR	^b^

AAR—aspartate aminotransferase-to-alanine aminotransferase ratio; ALT—alanine aminotransferase, AST—asparagine aminotransferase, APRI—aspartate aminotransferase-to-platelet ratio index, PLT—platelet count, FIB-4—Fibrosis-4 index; NLR—neutrophil-to-lymphocyte ratio; LMR—lymphocyte-to-monocyte ratio; PLR—platelet-to-lymphocyte ratio; NMR—neutrophil-to-monocyte ratio; CTP—Child-Turcotte-Pugh; MELD—Model for End-Stage Liver Disease, INR—international normalized ratio; ^a^—Child-Turcotte-Pugh (CTP) score consists of three objective symptoms of liver failure (serum bilirubin and albumin concentrations, prothrombin time) and two subjective symptoms (presence of ascites and encephalopathy). The CTP score ranges between 5 and 15 points, wherein three classes are distinguished: class A (5 to 6 points), class B (7 to 9 points), and class C (10 to 15 points). ^b^—9.57×ln(creatinine) ++3.78×ln(total bilirubin) ++11.2×ln(INR)+6.43; creatinine and bilirubin concentrations are in mg/dL. The computed score is rounded to an integer. If any of the laboratory parameters are lower than 1.0, it should be set to 1.0 for input for the formula to avoid generating a negative score. The maximum Model for End-Stage Liver Disease (MELD) score is 40. Any results higher than 40 are adjusted to 40 [13].

**Table 2:** The bacteria identified in diagnosed infections.

Infection Type	Infectious Agent	Number of Cases (% of All Infections)
UTI	*Escherichia coli* *Klebsiella pneumoniae* *Enterococcus faecalis*	32 (53.3%) 16 (26.7%) 4 (6.7%)
Pneumonia	*Mycobacterium tuberculosis* *Citrobacter freundi* *Candida albicans*	1 (1.7%) 1 (1.7%) 1 (1.7%)
Sepsis	*Staphylococcus aureus* *Coagulase-negative staphylococci* *Escherichia coli* *Streptococcus group B*	3 (5.0%) 2 (3.3%) 1 (1.7%) 1 (1.7%)
SBP	*Escherichia coli* *Enterobacter faecalis*	1 (1.7%) 1 (1.7%)
Infected post-operative wound	*Staphylococcus epidermidis*	1 (1.7%)
Abscess of subcutaneous tissue	*Escherichia coli*	2 (3.3%)

UTI—urinary tract infection; SBP—spontaneous bacterial peritonitis.

**Table 3:** Median and lower-upper quartile range of laboratory and clinical results in two groups of patients: with and without infection.

Parameter	Presence of Infection (*n* = 60)	Lack of Infection (*n* = 149)	*p*
NLR	2.45 (1.45–6.00)	1.85 (1.30–3.01)	0.024 ^a^
LMR	1.43 (1.06–2.01)	2.59 (1.73–3.84)	<0.001 ^c^
PLR	91.5 (58.8–118.6)	73.2 (50.8–99.6)	0.047 ^a^
NMR	5.00 (3.64–7.52)	4.87 (3.72–7.33)	0.988
Monocytes [G/L]	0.70 (0.42–1.02)	0.54 (0.36–0.75)	0.011 ^a^
AAR	1.88 (1.41–2.49)	1.57 (0.98–2.01)	0.014 ^a^
Modified APRI	15.86 (7.82–30.85)	12.18 (6.58–31.76)	0.755
FIB-4	6.25 (2.99–10.72)	6.37 (3.30–12.03)	0.960
CRP [mg/L]	30.7 (9.2–40.6)	6.5 (2.1–29.6)	0.020 ^a^
CTP	10 (8–11)	8 (6–9)	<0.001 ^c^
MELD	15 (12–22)	13 (10–17)	0.006 ^b^

^a^—*p* < 0.05, ^b^—*p* < 0.01, ^c^—*p* < 0.001. NLR—neutrophil-to-lymphocyte ratio; LMR—lymphocyte-to-monocyte ratio; PLR—platelet-to-lymphocyte ratio; NMR—neutrophil-to-monocyte ratio; AAR—aspartate aminotransferase-to-alanine aminotransferase ratio; APRI—aspartate aminotransferase-to-platelet ratio index; FIB-4—Fibrosis-4 index; CRP—C-reactive protein; CTP—Child-Turcotte-Pugh; MELD—Model for End-Stage Liver Disease.

**Table 4:** Area under the curve (AUC), cutoff values, Se, Sp, positive predictive value (PPV), and negative predictive value (NPV) for statistically significant parameters.

Parameter	AUC	*p*	Cut-Off	Se	Sp	PPV	NPV
NLR	0.606	0.033 ^a^	3.96	0.434	0.860	0.561	0.787
LMR	0.715	<0.001 ^c^	2.06	0.676	0.758	0.899	0.424
Monocytes	0.648	0.010 ^a^	0.91	0.455	0.867	0.517	0.835
AAR	0.610	0.013 ^a^	2.11	0.417	0.797	0.472	0.759
CRP	0.685	0.010 ^a^	9.2	0.760	0.600	0.613	0.750
CTP	0.687	<0.001 ^c^	9.0	0.702	0.639	0.485	0.816
MELD	0.629	0.005 ^b^	18	0.439	0.783	0.500	0.738

^a^—*p* < 0.05, ^b^—*p* < 0.01, ^c^—*p* < 0.001. NLR—neutrophil-to-lymphocyte ratio; LMR—lymphocyte-to-monocyte ratio; AAR—aspartate aminotransferase-to-alanine aminotransferase ratio; CRP—C-reactive protein; CTP—Child-Turcotte-Pugh; MELD—Model for End-Stage Liver Disease.

**Table 5:** Odds ratio for selected predictors.

	Univariate				Multivariate			
Predictor	Odds Ratio	CI	*p*	Nagelkerke’s R^2^	Odds Ratio	CI	*p*	Nagelkerke’s R^2^
LMR	0.15	0.06–0.37	<0.001 ^c^	0.199	0.22	0.08–0.64	0.005 ^b^	0.251
MONO	5.42	2.23–13.15	<0.001 ^c^	0.144				
NLR	4.38	2.09–9.17	<0.001 ^c^	0.116				
CRP	3.86	1.24–12.04	0.020 ^a^	0.133				
CTP	3.17	1.52–6.62	0.002 ^b^	0.090	2.77	1.01–7.60	0.048 ^a^	
MELD	3.18	1.54–6.56	0.002 ^b^	0.077				
AAR	2.68	1.39–5.18	0.003 ^b^	0.060				

^a^—*p* < 0.05, ^b^—*p* < 0.01, ^c^—*p* < 0.001.
